# The SAAP pipeline and database: tools to analyze the impact and predict the pathogenicity of mutations

**DOI:** 10.1186/1471-2164-14-S3-S4

**Published:** 2013-05-28

**Authors:** Nouf S Al-Numair, Andrew CR Martin

**Affiliations:** 1 Institute of Structural and Molecular Biology, Division of Biosciences, University College London, Darwin Building, Gower Street, London WC1E 6BT, UK

## Abstract

**Background:**

Understanding and predicting the effects of mutations on protein structure and phenotype is an increasingly important area. Genes for many genetically linked diseases are now routinely sequenced in the clinic. Previously we focused on understanding the structural effects of mutations, creating the SAAPdb resource.

**Results:**

We have updated SAAPdb to include 41% more SNPs and 36% more PDs.

Introducing a hydrophobic residue on the surface, or a hydrophilic residue in the core, no longer shows significant differences between SNPs and PDs. We have improved some of the analyses significantly enhancing the analysis of clashes and of mutations to-proline and from-glycine. A new web interface has been developed allowing users to analyze their own mutations. Finally we have developed a machine learning method which gives a cross-validated accuracy of 0.846, considerably out-performing well known methods including SIFT and PolyPhen2 which give accuracies between 0.690 and 0.785.

**Conclusions:**

We have updated SAAPdb and improved its analyses, but with the increasing rate with which mutation data are generated, we have created a new analysis pipeline and web interface. Results of machine learning using the structural analysis results to predict pathogenicity considerably outperform other methods.

## Background

The explosion in the availability of mutation data, resulting from the application of SNP chips [1] and next-generation sequencing [2] has led to a huge demand to analyze and predict the effects of mutations. The genes for many genetically linked diseases are now routinely sequenced in the clinic.

While a mutation is defined as 'any change in the DNA', most work has focused on studying 'Single Nucleotide Variations' (SNVs). Broadly these can be classified into Single Nucleotide Polymorphisms (SNPs) and pathogenic deviations (PDs). SNPs which, if strictly defined, occur in at least 1% of a normal population, are estimated to occur once every 100-300 bases in the human genome [3], giving rise to subtle phenotypic variation without causing major deleterious phenotypic changes; PDs occur at much lower frequencies and are causative of disease.

In reality, SNVs form a spectrum from completely silent SNPs at one end, to 100% penetrance, Mendelianly inherited PDs at the other end. In between, SNVs show partial penetrance; that is, only a fraction of individuals having the mutation show altered phenotype and this can be influenced by the presence of other mutations and/or environmental factors.

To date, most effort has gone into understanding the effects of missense SNVs that lead to changes in protein sequence. We use the term 'Single Amino Acid Polymorphism' (SAAP) to refer to such amino acid changes whatever the frequency and resulting phenotype of the mutation. More than a dozen groups have devised methods to analyze the effects a given SAAP will have and in some cases attempt to predict whether the mutation will have a deleterious effect on phenotype [4-15]. However, the best known methods are SIFT [16] (an evolutionary method which calculates a sophisticated residue conservation score from multiple alignment) and PolyPhen-2 [17] which uses machine learning on a set of eight sequence- and three structure-based features. A recent addition to the set of tools is Condel [18], a consensus predictor which makes use of SIFT, PolyPhen-2 and MutationAssessor [12]. Condel significantly outperforms any of its component predictors. Until recently, rather than trying to predict whether a given SAAP will result in a deleterious phenotype, our focus has been on trying to understand the effects that mutations have on protein structure, comparing these effects in SNPs (that is non-pathogenic mutations) and PDs. Our approach has been to map SAAPs onto protein structure and to perform a rule-based analysis of the likely structural effects of these mutations in order to 'explain' the functional effect (if any) of the mutation. Since we map mutations to structure, we only consider mutations in proteins for which a structure has been solved. Data resulting from the analysis of SNPs and PDs have been collected into a relational database and made available over the web in the resource SAAPdb [19] (http://www.bioinf.org.uk/saap/db/).

In this paper we describe (i) an update of the data in SAAPdb, (ii) enhancements to methods used to analyze the structural impact of SNPs, (iii) a new web interface allowing the analysis of new mutations and (iv) results of the application of machine learning to predict the phenotypic effects of mutations based on our structural analyses.

## Results and discussion

### SAAPdb update

**Figure 1:**
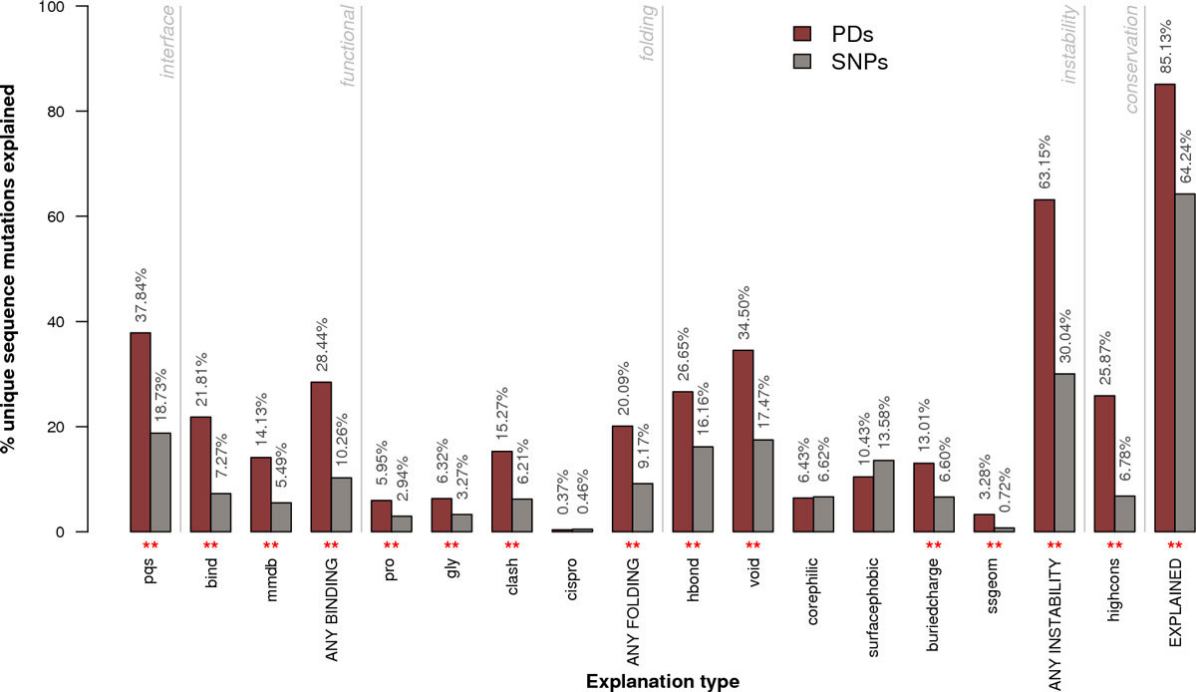
**PQS **Residuein a contact with a different protein chain or ligand, according to the PQS server;**** Bind ****Residue in a contact with a different protein chain or ligand, according to PDB data; ****MMDB ****Residue in a contact with a ligand, according to the MMDB server;**** Gly ****Mutation from glycine, introducing unfavourable torsion angles; ****Pro ****Mutation to proline, introducing unfavourable torsion angles;**** Cispro ****Mutation from cisproline, introducing unfavourable torsion angle; ****Clash ****Mutation introducing a steric clash with an existing residue; ****Void ****Mutation introducing a destabilizing void >275Å^3 ^in the protein core;**** Hbond ****Mutation disrupting a hydrogen bond; ****CorePhilic ****Introduction of a hydrophilic residue in the protein core;**** SurfacePhobic ****Introduction of a hydrophobic residue on the protein surface; ****BuriedCharge ****Mutation causing an unsatisfied charge in the protein core; **SSgeom** Mutation disrupting a disulphide bond; **HighCons** Residue has conserved sequence;**** EXPLAINED ****Any of the above categories. In addition, we look at whether a residue annotated as functionally relevant by UniProtKB/SwissProt; Asterisks indicate a significant result (two where *p *< 0.01 and one where *p *< 0.05) calculated as described in Hurst *et al*. [19].

Considerable effort has been made to improve the code for updating SAAPdb. A summary of the datasets comparing the old and new builds of the database is shown in Table 1, while Figure 1 (which can be compared with the Hurst *et al*. paper [19]) shows a comparison of structural effects seen for SNPs and PDs. Other sources of mutation data have been considered including HGMD and SwissProt Variants (SwissVar). However HGMD data are only available to registered users meaning that we have not been able to reproduce their data in our database and SwissVar is not terribly reliable in annotation of disease status. For example, known PDs in G6PD are annotated as 'Natural Variants' of Unclassified disease status. Other locus specific mutation databases (LSMBDs) can easily be added [20], but as explained below, we have now implemented SAAPdap, a pipeline version of the system allowing users to analyze novel mutations, which we now regard as our primary resource.

**Table 1:** Number of distinct mutations from different sources that have been mapped to protein sequence and included in SAAPdb


**Data source**	**Previous build**	**Current build**

**SNP**s		
dbSNP	34342	48452
**PD**s		
OMIM	7249	9339
ADABase Adenosine DeAminase deficiency	30	38
Hamsters The Haemophilia A Mutation, Structure, Test	530	628
and Resource		
IARC-p53-Germline Tumor Protein 53 gene germline	95	138
mutation in familial cancers		
IARC-p53-Somatic Tumor Protein 53 gene somatic mu-	617	1368
tations in sporadic cancers		
G6PD Glucose-6-Phosphate Dehydrogenase	170	170
OTC Ornithine TransCarbamylase (OTC)	148	217
SODdb SuperOxide Dismutase 1	96	125
ZAP70Base Zeta-chain-Associated Protein kinase 70	5	5
Kinbase Somatic protein kinase driver mutations	66	66
Kinbase Somatic protein kinase passenger mutations	66	66
LDLR Low Density Lipoprotein Receptor	516	515
PAHdb Human Phenylalanine Hydroxylase gene	0	337
STAT3 Signal Transducer and Activator of Transcription	0	47
3		
Total PDs	9588	13059

Since we map mutations to protein structure and therefore require a structure to be solved of the protein of interest, we are not able to analyze all mutations. Of the amino acid mutations in OMIM, we are only able to map approximately 57% to structure, while only approximately 22% of 'valid' SNPs from dbSNP, which result in an amino acid change, map to structure. Consequently the coverage of our analysis is currently somewhat limited, but clinically relevant proteins tend to be key targets for structural studies, so we expect this figure to improve. Where multiple structures have been solved, we analyze the effects of the mutations in all available structures.

In summary, the number of SNPs in the database has risen by 41% and the number of PDs by 36% (including two new sources of mutation data). The comparison of structural effects between SNPs and PDs shows the same trends as in the previous analysis, but the 'surfacephobic' (introducing a hydrophobic residue onto the surface) and 'corephilic' (introducing a hydrophilic residue into the core) analyses no longer show significant differences between SNPs and PDs.

### Analysis enhancements

In SAAPdb, all assignments of structural effects are Boolean -- that is, any mutation either does, or does not, have a given effect. While Boolean assignment is appropriate in some cases (for example, a residue either is, or is not, annotated as a feature in UniProtKB/SwissProt), in other cases, it relies on some cutoff (for example, energy, void volume, hydrophobicity difference) as described previously [19,21-23].

We found that assigning a mutation as (not) having a structural effect is very sensitive to precise structural details; where multiple structures are available for the same protein, one structure may indicate that a mutation has a value just below a cutoff while another structure has a value just above. Wherever appropriate, we have now implemented real-number scores or pseudo-energies for each effect. In particular, we have enhanced the analysis of clashes and torsion angles to provide energy values.

#### Clashes

In analyzing clashes, our previous work defined a damaging clash as any sidechain that has at least 3 van der Waals overlaps (of any degree) with other atoms [19]. We now perform a more complete energy calculation incorporating Lennard-Jones and torsion energies using CHARMM [24] parameters:

(1) $$E=\left(\frac{A}{r^{12}}-\frac{B}{r^{6}}\right)+k\left(1+\text{cos}(n\psi +\phi \text{)}\right)$$

**Figure 2:**
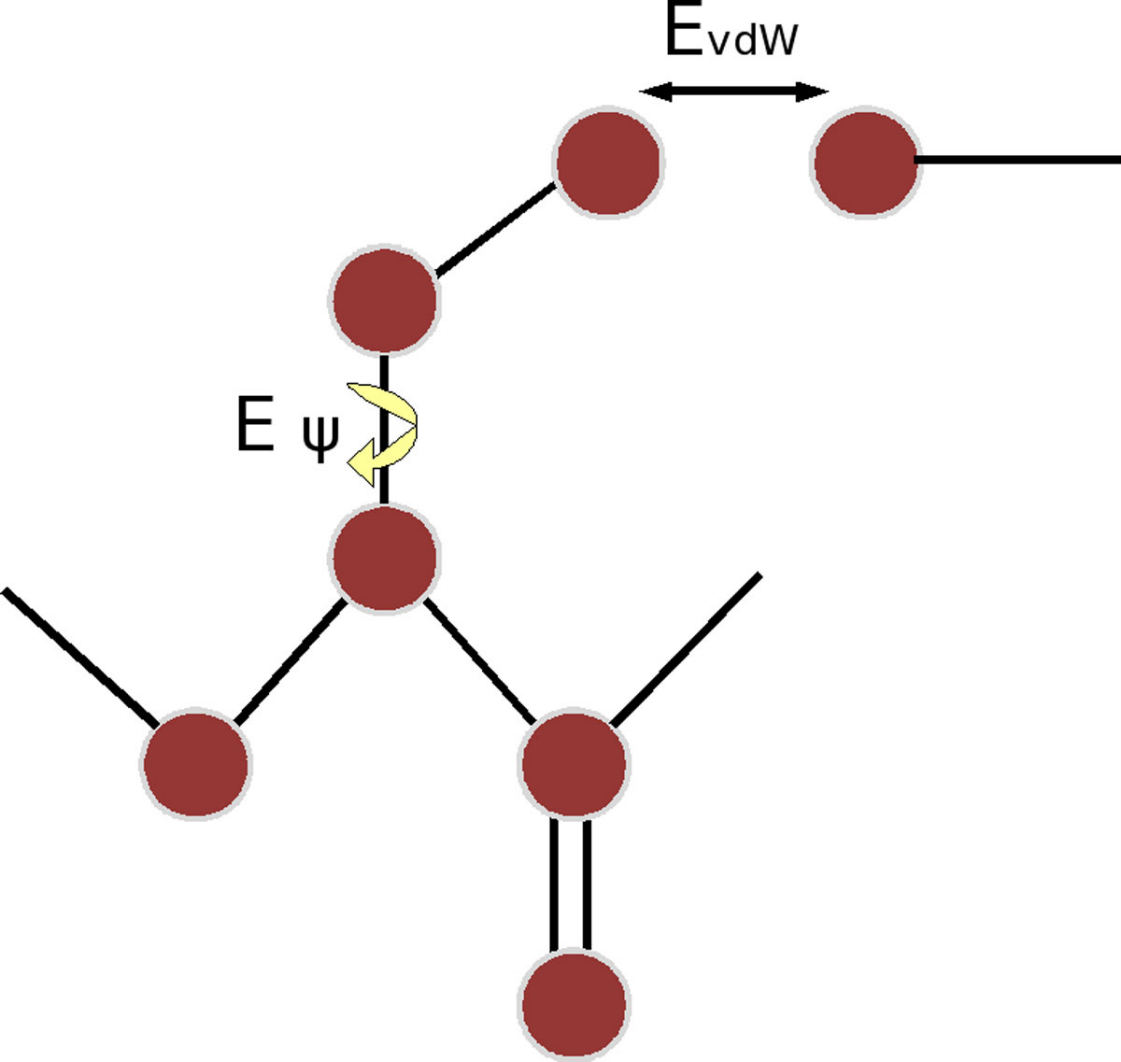
**Schematic indicating the two new terms used in evaluation of clashes**. *E_vdW _*is the van der Waals energy evaluated using a standard Lennard-Jones potential while *E_ψ _*is a torsion energy.

**Figure 3:**
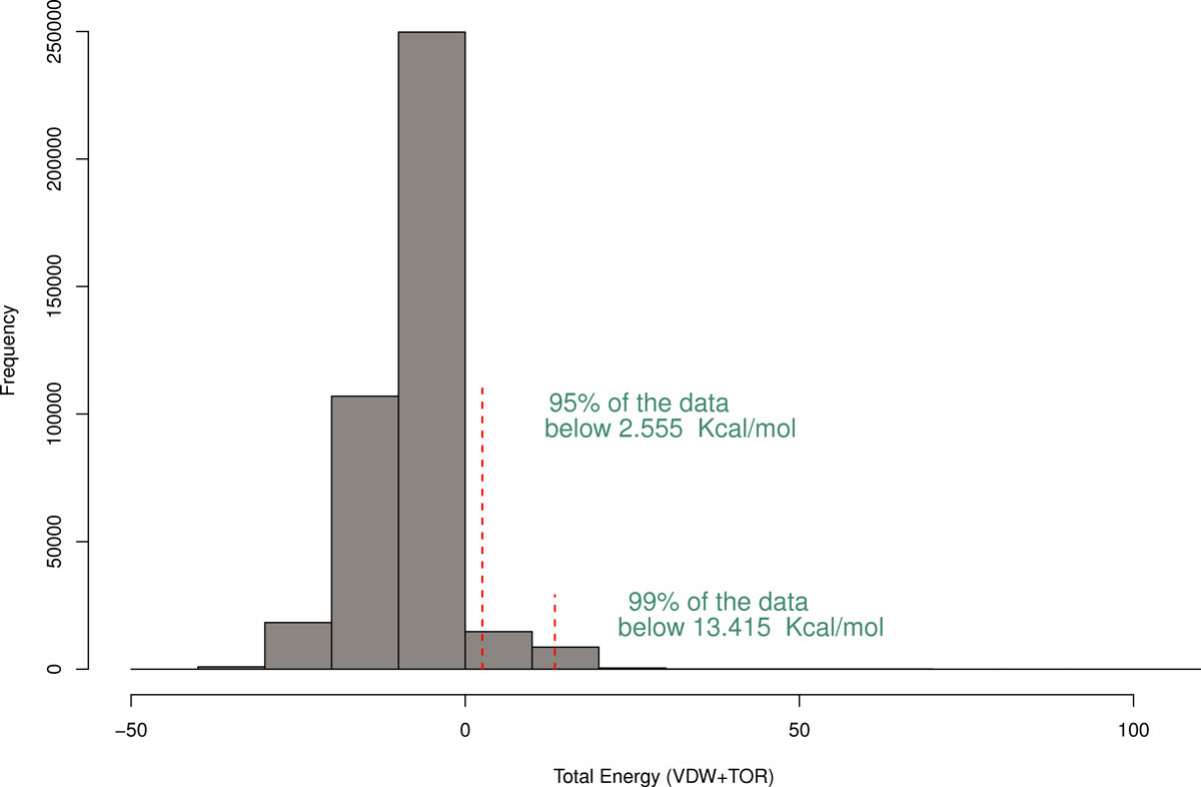
**Distribution of sidechain clash energies calculated according to Equation 1 for high resolution structures amongst CATH O-representatives**.

This accounts for any clashes between atoms of the sidechain being replaced with its surroundings, together with preferences for staggered conformations (see Figure 2). Testing the new method on 400,000 residues from CATH O-representatives (domains having no more than 65% sequence identity) of high resolution (**<**2.5Å) shows that 99% of sidechains have an energy of **<**13.4 kcal/mol (see Figure 3).

**Figure 4:**
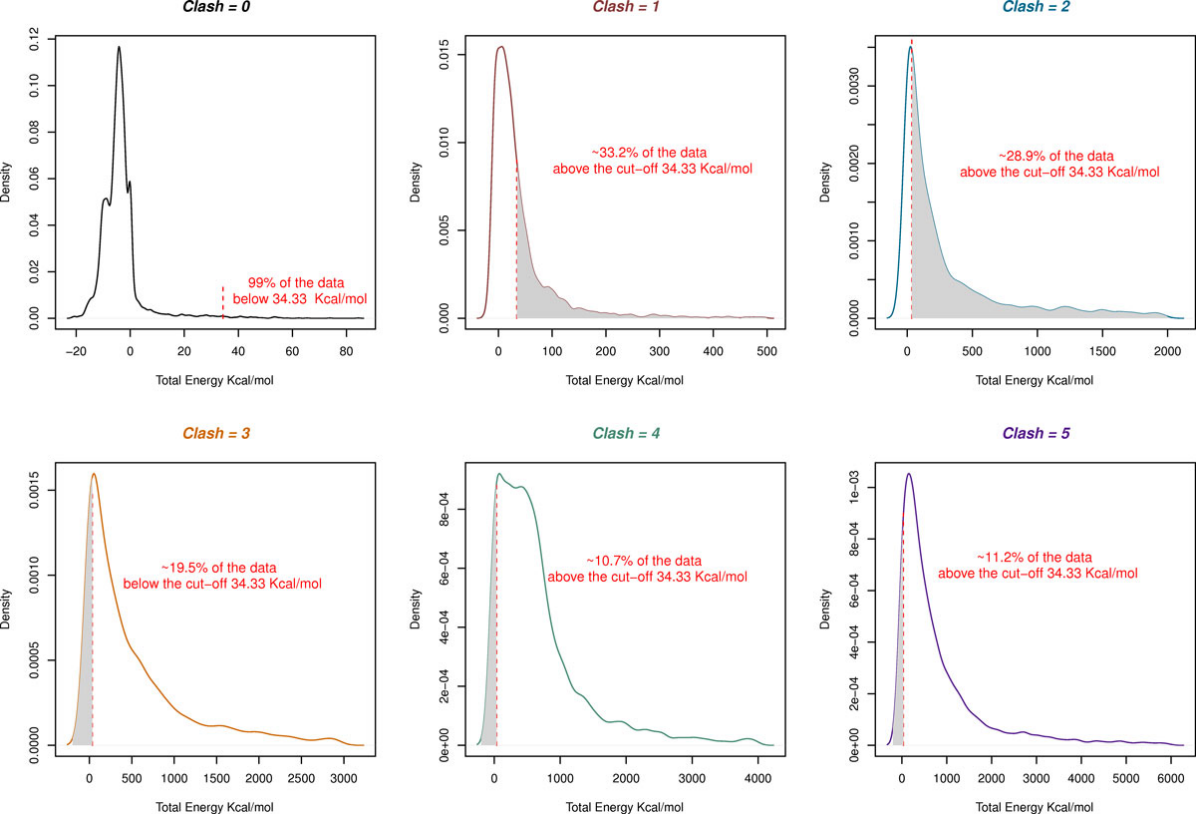
**Distribution of energies calculated according to Equation 1 for sidechain replacements classified as making 0-5 clashes using the old (Boolean) method**. In the old method, 0, 1, or 2 clashes were considered not to form a bad clash while 3 or more clashes were considered to be a bad clash. In each plot, the shaded area shows those residues that were mis-classified according to the new energy-based criterion.

Using the new energy evaluation we went on to analyze how the old clash method performed. In the old method, no account was made of the degree of clash -- overlaps of 0.01Å or of 1.0Å were treated the same. Figure 4 shows the energy distribution for sidechain replacements considered to make 0-5 clashes by the old method. Looking at sidechain replacements that made no clashes using the old method (Figure 4, panel 1), we see that 99% of the data have an energy below 34.33 kcal/mol using the new energy-based method. Panels 2 and 3 show cases evaluated as making one or two clashes using the old method. Using 34.33 kcal/mol as an energy cutoff, these graphs indicate that 33.2% and 28.9% of potentially damaging clashes (shaded regions in panels 2 and 3 respectively) were not detected using the old method. Panels 4, 5 and 6 show the energy distributions for sidechain replacements having 3-5 clashes by the old method which would have been classified as damaging. However, using the new method, 19.5%, 10.7% and 11.2% of cases (shaded regions in panels 4, 5 and 6 respectively) have energies below the threshold and are therefore unlikely to be damaging.

Overall, approximately 32% of mutations previously classified as not clashing are now found to clash while approximately 15% of mutations previously classified as clashing are now found to have only minor clashes which could be relieved by very slight movements in the structure.

#### Glycine and proline mutations

Glycine and proline are the 'structural' amino acids which show an unusual Ramachandran distribution. Because glycine has no sidechain, it is able to access a wider range of phi/psi combinations while the cyclic sidechain of proline restricts the available phi angles. Consequently, backbone conformational changes may be necessary to accommodate mutations from-glycine or to-proline.

**Figure 5:**
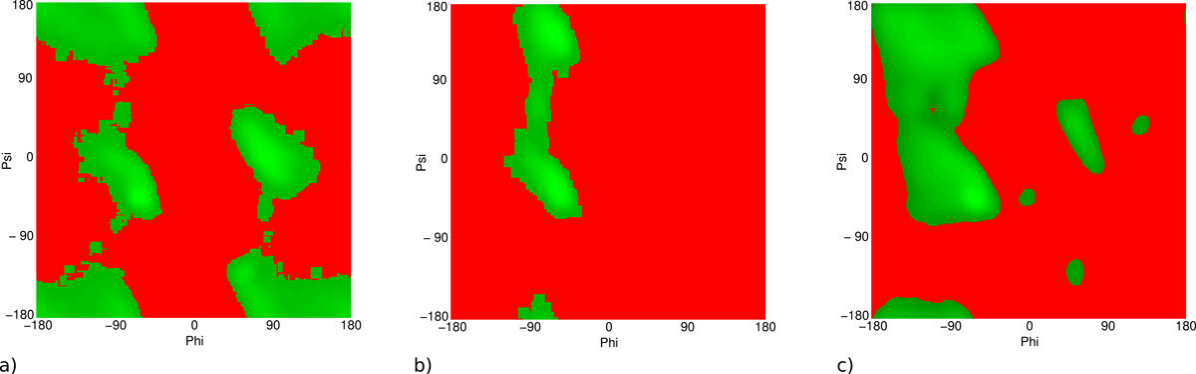
**Ramachandran plots generated from high resolution structures**. a) glycine, b) pro-line, c) other. Favoured regions are shown in progressively paler green while disfavoured regions are shown in red.

Previously, we used a very simple set of allowed boundaries for backbone phi/psi angles. We have now developed a pseudo-energy potential based on Ramachandran plots. A non-redundant set (sequence identity **<**25%) of high resolution protein domains (resolution **≤ **1.8Å, R-Factor **≤**0.3) was selected from CATH and Ramachandran plots were generated on a 1-degree grid for proline, glycine and 'other' amino acids. The plot was smoothed by averaging each of the cells with its eight neighbours (Figure 5). For each cell, we can then calculate:

(2) $$E=-\text{log}\left(\frac{\text{obs}}{\text{exp}}\right)$$

where 'obs' is the (smoothed) observed number of residues with a given phi/psi combination while 'exp' is the expected number, calculated as the total number of observations divided by the number of cells. A threshold energy was calculated for each plot based on 1% of observations in high quality non-redundant structures having a worse energy.

### SAAP data analysis pipeline (SAAPdap)

SAAPdb was designed as a regularly updated pre-calculated resource. However, it has proved very difficult to maintain and changes in licensing of OMIM data mean that we may no longer be able to use this as our primary source of PDs. In addition, with the increasing routine use of high-throughput sequencing methods to detect mutations, more and more people want to be able to analyze their own mutations.

Consequently we believe the value of SAAPdb has diminished and have now implemented SAAPdap (Single Amino Acid Polymorphism Data Analysis Pipeline). This is a complete rewrite of the mutation analysis software in SAAPdb using a plugin architecture and making use of the new non-Boolean analyses. While we still indicate whether a mutation is likely to have a detrimental effect on structure using cutoff values, we also provide continuous values for each of the analyses.

**Figure 6:**
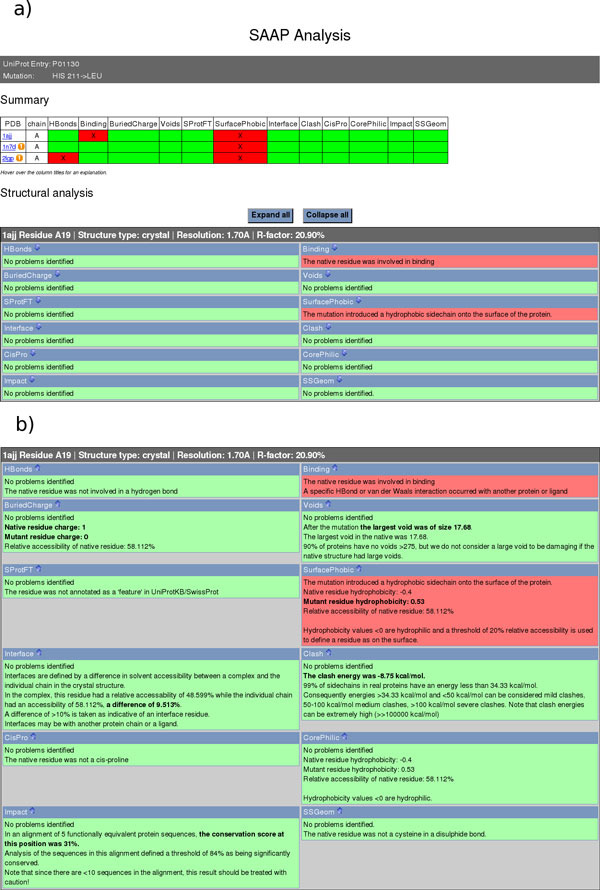
**Results pages from the new SAAPdap pipeline**. a) Summary and brief structural reports -- hovering over any of the titles brings up a box to explain the meaning of the effect; b) Expanded view of full structural analysis.

Results from the SAAPdap pipeline are presented as shown in Figure 6a. Results are summarized at the top where the effects on each structure to which the mutation maps are shown. Below, the analyses of structural effects on each structure are presented and these can then be expanded to provide more detail on the analyses as shown in Figure 6b. Analysis descriptions are much more comprehensive than was the case in SAAPdb to make the results easier to understand.

We have now implemented a web interface to allow users to enter mutations for analysis. Because some of the analyses (especially the analysis of voids) are quite time consuming (taking several minutes), the web interface makes use of AJAX (Asynchronous JavaScript And XML) to update the user with the progress of the analysis. The submission page is available at http://www.bioinf.org.uk/saap/dap/.

### Predicting damaging mutations (SAAPpred)

The data in SAAPdb (Figure 1) show clear differences in the sequence and structural characteristics of SNPs and PDs: PDs have additional, and more severe, structural effects. Thus there is a clear signal that can be used to predict the pathogenicity of a novel mutation.

In preliminary experiments, we used a balanced set of mutation data from SAAPdb with Random Forests implemented in Weka obtaining an accuracy (Acc) of 0.935 and a Matthews Correlation Coefficient (MCC) of 0.871 (based on 10-fold cross-validation). The balanced dataset consisted of 30,500 SNPs mapped to unique PDB structures (see Table 2) and processed without any errors, and a random selection of 30,500 PDs (mapped to unique PDBs). Where several structures are available for a mutated residue, each is used as an independent data point in training the machine learning. While the 10-fold cross-validation in Weka ensures that there is no direct overlap between the training and test sets, in these preliminary experiments, there may be some 'structural' overlap -- in other words, for a given mutation in the test set, the same mutation in a different PDB file of the same protein may be present in the training set.

**Table 2:** Breakdown of the number of mutations in SAAPdb and their mapping to structure.

Number of Mutations	PDs	SNPs
Mapped to UniProtKB/SwissProt	13,059	48,452
Mapped to PDB	6,527	17,915
Mapped to multiple PDBs	202,566	33,369
Mapped to multiple Chains	405,497	45,699

In some cases, several hundred structures are available (e.g. haemoglobin, carbonic anhydrase, prthrombin, transthyretin, insulin, CDK2, lysozyme) and, on average there are approximately two copies of each chain in each PDB file.

González-Pérez and López-Bigas [18], report that well known individual methods (SIFT, PolyPhen2, Logre [25], MAPP [26] and MutationAssessor) give accuracies between 0.690 and 0.771 evaluated on the HumVar dataset developed for PolyPhen2. Their consensus method (Condel) gives an accuracy of 0.882. While our preliminary value of 0.935 is considerably better, we are using a different dataset.

**Figure 7:**
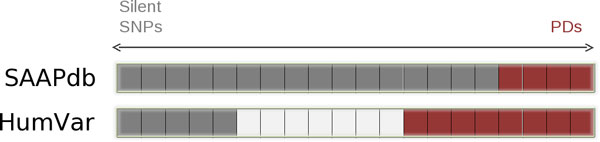
**The penetrance of a mutation lies on a scale between 'True SNPs' which show no phenotypic effect at one extreme to Mendelianly inherited PDs with 100% penetrance at the other**. In SAAPdb, we use a very conservative definition of PDs, but a rather wide definition of SNPs. In contrast, HumVar uses a somewhat broader definition of PDs, but a much more conservative definition of SNPs and does not consider mutations that lie in the middle.

However, having trained on SAAPdb, if we test on 1540 SNPs and 7182 PDs from the HumVar dataset that mapped to structure we obtain Acc = 0.446, MCC = 0.135 --essentially a random prediction. This appears to be because of the different definition of the 'boundary' between SNPs and PDs. As stated above, SNVs form a spectrum from completely silent SNPs at one end, to 100% penetrance, Mendelianly inherited PDs at the other end. As shown in Figure 7, different datasets use different thresholds to separate the data into two sets or may consider only the extremes. Prediction of the extremes may appear to be a trivial problem, but this is not always the case -- some damaging mutations are very hard to predict. HumVar uses a broader definition of PDs than the SAAPdb data; in contrast, the SAAPdb definition of SNPs is rather wide (anything in dbSNP not annotated as being involved in disease) while the definition in HumVar enforces the requirement that SNPs are present in at least 1% of a normal population. Since, in this experiment, we use very different definitions for the training and testing, it is not surprising that we obtain poor performance. We considerably over-predict SNPs, consistent with our broader definition of SNPs in the SAAPdb dataset.

Consequently, for the final version of SAAPpred, we both trained and tested our method on the HumVar dataset (using 10-fold cross-validation). HumVar (2011/12) contains 22,196 deleterious mutations and 21,151 neutral mutations of which 7,182 and 1,540, respectively, can be mapped to structure. Consequently, to obtain a balanced dataset, only 3,080 mutations (equal numbers of deleterious and neutral) can be used. Ten runs were performed, each of which used all 1,540 neutral mutations with a random selection of 1,540 deleterious mutations from the total of 7,182. Results from the ten runs were then averaged. In each run, to avoid the 'structural overlap' between the training and testing data during cross-validation (which was present in the preliminary experiments with SAAPdb data), the mutations were split into 10 sets of approximately the same size. Each of these 10 sets in turn was chosen as a test set. The remaining 9 sets were used for training by randomly drawing balanced datasets of different sizes from the mutations as mapped to protein chains (see Table 2). This manual cross-validation ensures that there are no cases of the same mutation in the training and test sets but from different PDB chains.

**Figure 8:**
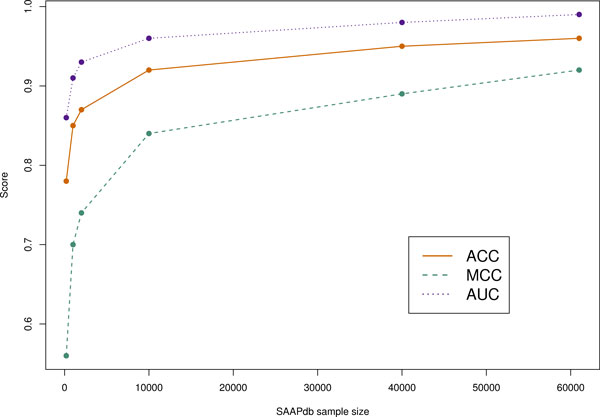
**Performance of the machine learning method trained on different sized sets of data from SAAPdb**. In each case, a balanced dataset of the required size was extracted at random from the SAAPdb dataset of mutations mapped to protein chains (Table 2) and random forests were trained and tested using 10-fold cross-validation. The graph clearly shows that performance drops as the dataset size decreases, showing a marked drop in performance with datasets below 10,000 samples in size (5,000 SNPs and 5,000 PDs).

As expected, the performance (Acc = 0.846, MCC = 0.692) is rather worse than with the SAAPdb data, simply because the size of the HumVar dataset that can be mapped to structure is much smaller than the SAAPdb dataset. Figure 8 shows the effect of dataset size on training and testing using subsets of the SAAPdb data. The same procedure described above was used to avoid structural overlap between training and testing sets during cross-validation. The graph clearly shows that the smaller datasets perform considerably worse.

Nonetheless, our results from training and testing on HumVar mutations that map to structure considerably outperform other well-known individual methods including SIFT and PolyPhen2 as reported by González-Pérez and López-Bigas [18] (Accuracies between 0.690 and 0.771). However these results are still not directly comparable with the other methods as those methods are evaluated on the complete HumVar dataset and it may be argued that the subset of mutations for which structures are available somehow outperform those for which structures are not available in these other methods. For example, PolyPhen2 makes limited use of structural data where these are available.

Consequently, we have evaluated the performance of PolyPhen2, SIFT and MutationAssessor using balanced datasets (1451 neutral mutations and ten random selections of 1451 deleterious mutations) used to assess the performance of our method. (Note that we could only use 1451 rather than 1540 mutations since the remaining 89 PDs failed in at least one of the other predictors.) In fact this gives a significant advantage to PolyPhen2 which is itself trained on HumVar leading to an overlap between the training and test set. It is not clear precisely what data are used to train SIFT; in their latest paper, Sim *et al*. [27]. state that SIFT was originally trained and tested on LacI, Lysozyme and HIV protease, and refer to the original SIFT papers, but they do not state whether the training has since been modified. MutationAssessor does not appear to use a training set *per se*.

Results are summarized in Table 3 where it can be seen that our method (SAAPpred) evaluated using 10-fold cross-validation (i.e. with no overlap between test and training sets) performs better than competing methods where there may be overlap between testing and training data. In particular, PolyPhen-2 was trained on the complete HumVar dataset from which our test set is extracted. If we allow overlap in our own set (the fairest comparison) then we outperform PolyPhen2 (the best of the competing methods) by an even larger margin.

**Table 3:** Performance of different prediction methods using a balanced dataset of mutations that map to structure extracted from HumVar.

Method	Cross-validated	MCC	Acc
SAAPpred	Yes	0.692	0.846
SAAPpred	No	0.894	0.944
PolyPhen2	No	0.572	0.785
SIFT	?	0.528	0.763
MutationAssessor	N/A	0.453	0.698

The values for the cross-validated assessment of SAAPpred were obtained from 10-fold cross-validation performed during the Weka training and used all 1540 SNPs from HumVar that mapped to structure with a random sample of 1540 of the 7182 PDs that mapped to structure. This was repeated 10-times and the results averaged. Non cross-validated results were performed by using a slightly smaller set of 1451 SNPs that mapped to structure and could be assessed by all the other methods together with a random sample of 1451 PDs that could be assessed by all methods. Again this was repeated 10-times and the results averaged. The non-cross-validated values for SAAPpred give the fairest comparison with PolyPhen2 which is trained on the HumVar dataset. It is unclear exactly what data were used in training the most recent version of SIFT so there may be some overlap between training and test sets while MutationAssessor has no training set *per se*.

While SAAPpred is clearly performing extremely well, we expect to be able to improve results further through feature selection (to help with the relatively small HumVar dataset size), feature combination (e.g. subtracting native void sizes from mutant void sizes) and feature normalization (e.g. taking the log of some feature values to improve the distribution of values). We also hope to develop methods to make more complete use of unbalanced datasets and intend to use our predictor as a component predictor of the consensus predictor Condel [18] which outperforms the other individual methods (Acc = 0.882).

## Conclusions

In conclusion, we have updated the data in SAAPdb, improved the analyses and integrated these into the new SAAPdap pipeline and web interface. It is intended that SAAPdap will replace SAAPdb (which has proved difficult to update regularly and reliably). The submission page for SAAPdap is available at http://www.bioinf.org.uk/saap/dap/.

We are currently working on new analyses that examine sequence differences at the DNA and RNA level. In addition to changes to the protein structure, mutations can have effects on expression, RNA splicing and RNA folding and stability [28-30].

Results of machine learning using the structural parameters calculated in SAAPdap considerably out-perform any other individual predictor and approaching the performance of the combined predictor, Condel. Future work will further optimize the performance of this method using feature selection, feature combination and feature normalization as well as exploiting strategies to make more complete use of unbalanced datasets. We will integrate our predictor as a component of Condel and expect performance to outperform the current Condel method.

While the coverage of our method is currently somewhat limited by the need for a structure of the protein, we plan to investigate the use of modelled structures. However, we currently don't know how well this will work given the detailed structural analysis (e.g. of hydrogen bonds) that our method performs. However clinically relevant proteins tend to be key targets for structural studies, and as more structures become available, the number of mutants mapped to structure will increase, improving the coverage of our method. In addition, more structural data will allow us to train the machine learning methods with more data. Consequently, as shown in Figure 8, we expect performance to increase further.

## Materials and methods

SNP data were extracted from the XML format dump of dbSNP [31] obtained from the NCBI. Non-synonymous, 'valid' human SNPs (i.e. those annotated with validation strings 'by frequency', 'by 2hit 2allele', or 'by hapmap'), were extracted and combined into a single XML file. Any mutations not annotated as having disease involvement were assumed to be neutral. PDs were obtained from Online Mendelian Inheritance in Man (OMIM, http://www.ncbi.nlm.nih.gov/omim/) and a number of locus-specific mutation databases ('LSMDBs') [20], see Table 1. All mutations were then mapped to protein sequences and thence to structure as described previously [19].

SAAPdb and SAAPdap perform fourteen analyses: **Interface: **(or **PQS:**) Residue is in an interface according to solvent accessibility criteria; **Binding: **Residue makes specific interactions with a different protein chain or ligand; **SprotFT: **Residue is annotated as functionally relevant by UniProtKB/SwissProt; **Clash: **Mutation introduces a steric clash with an existing residue; **Void: **Mutation introduces a destabilizing void in the protein core; **Cis-Proline: **Mutation from cis-proline, introducing an unfavorable omega torsion angle; **Glycine: **Mutation from glycine, introducing unfavorable torsion angles; **Proline: **Mutation to proline, introducing unfavorable torsion angles; **HBond: **Mutation disrupts a hydrogen bond; **Corephilic: **Introduction of a hydrophilic residue in the protein core; **Surfacephobic: **Introduction of a hydrophobic residue on the protein surface; **Buriedcharge: **Mutation causes an unsatisfied charge in the protein core; **SSgeometry: **Mutation disrupts a disulphide bond; **Impact: **Residue is significantly conserved. From these analyses (using software written in Perl and C) we derive 47 features that are used for machine learning. Random Forests (implemented in Weka [32]) were used for all predictions. Random Forests are ensemble classifiers that consist of multiple decision trees, each of which uses a random subset of the available features. The output of the predictor is the fraction of trees voting for the most popular class (in this case PD or SNP). Initial trials were performed using SAAPdb and HumVar data with 1000 trees and between 4 and 45 features per tree. 40 features performed best with SAAPdb while 4 features performed best with HumVar and these values were used for building the final machine learning models. Data are stored in a PostgreSQL relational database.

### Note added in proof

An additional predictor FATHMM (http://fathmm.biocompute.org.uk/) has become available since this work was completed (Shihab HA, Gough J, Cooper DN, Stenson PD, Barker GLA, Edwards KJ, Day INM, Gaunt, TR. (2013). Predicting the Functional, Molecular and Phenotypic Consequences of Amino Acid Substitutions using Hidden Markov Models. Hum. Mutat., 34:57-65). Evaluation of FATHMM on the same dataset shows a performance of ACC=0.836, MCC=0.671. While approaching our cross-validated performance, it is likely that some of the HumVar data were included in training FATHMM.

## Competing interests

The authors declare that they have no competing interests.

## Authors' contributions

NSAN updated the SAAPdb code and data, wrote some of the enhanced analysis methods, performed the machine learning and all testing, drafted the paper and created the figures. ACRM implemented SAAPdap and the remaining enhanced analysis methods, revised the paper and contributed to the machine learning.

## Declarations

The publication costs for this article were funded by the above grant.

This article has been published as part of *BMC Genomics *Volume 14 Supplement 3, 2013: SNP-SIG 2012: Identification and annotation of SNPs in the context of structure, function, and disease. The full contents of the supplement are available online at http://www.biomedcentral.com/bmcgenomics/supplements/14/S3
